# Supplementary material for: Autoregulation of three yeast ribosomal protein genes by splicing inhibition
Source: G3 (Bethesda). 2026 Mar 30;16(6):jkag083. doi: 10.1093/g3journal/jkag083 (PMC13232506; doi:10.1093/g3journal/jkag083)
Supplement: jkag083_Supplementary_Data [file jkag083_supplementary_data.zip › Supplemental_Material_G3-2026-406531.pdf]

Supplemental Figures

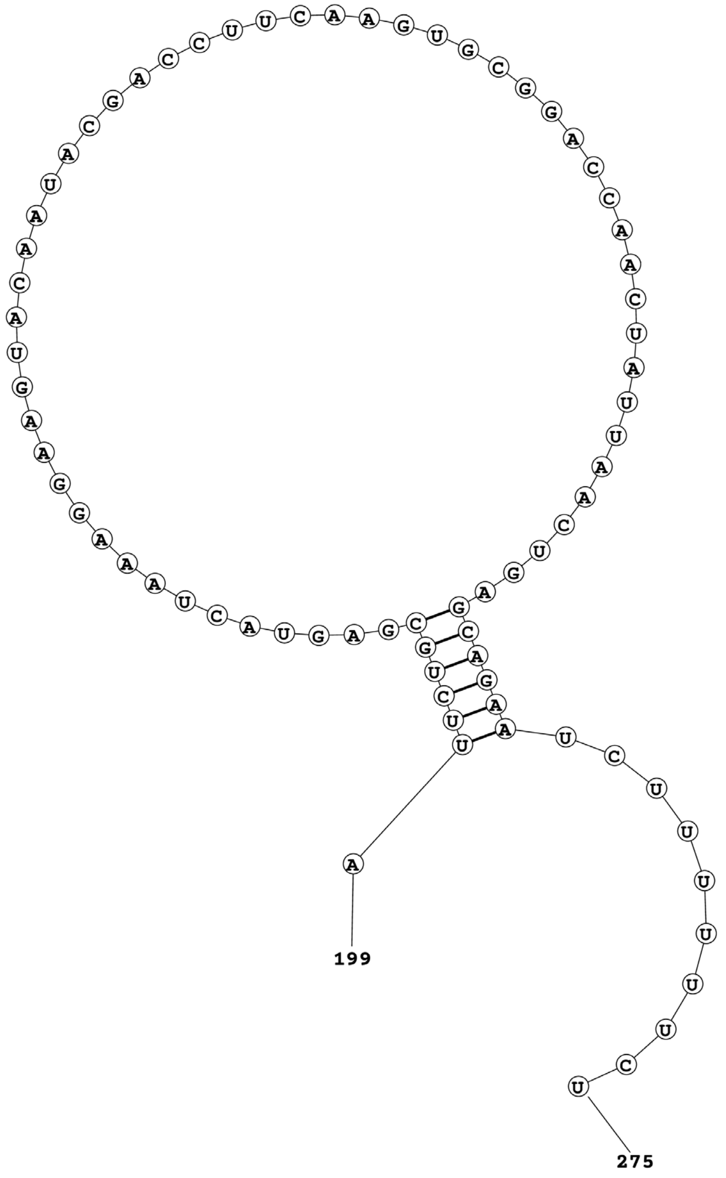

Supplemental Figure S1. Predicted Hooks structure for *RPL18B* intron. Numbering is for positions within the 432-base intron.

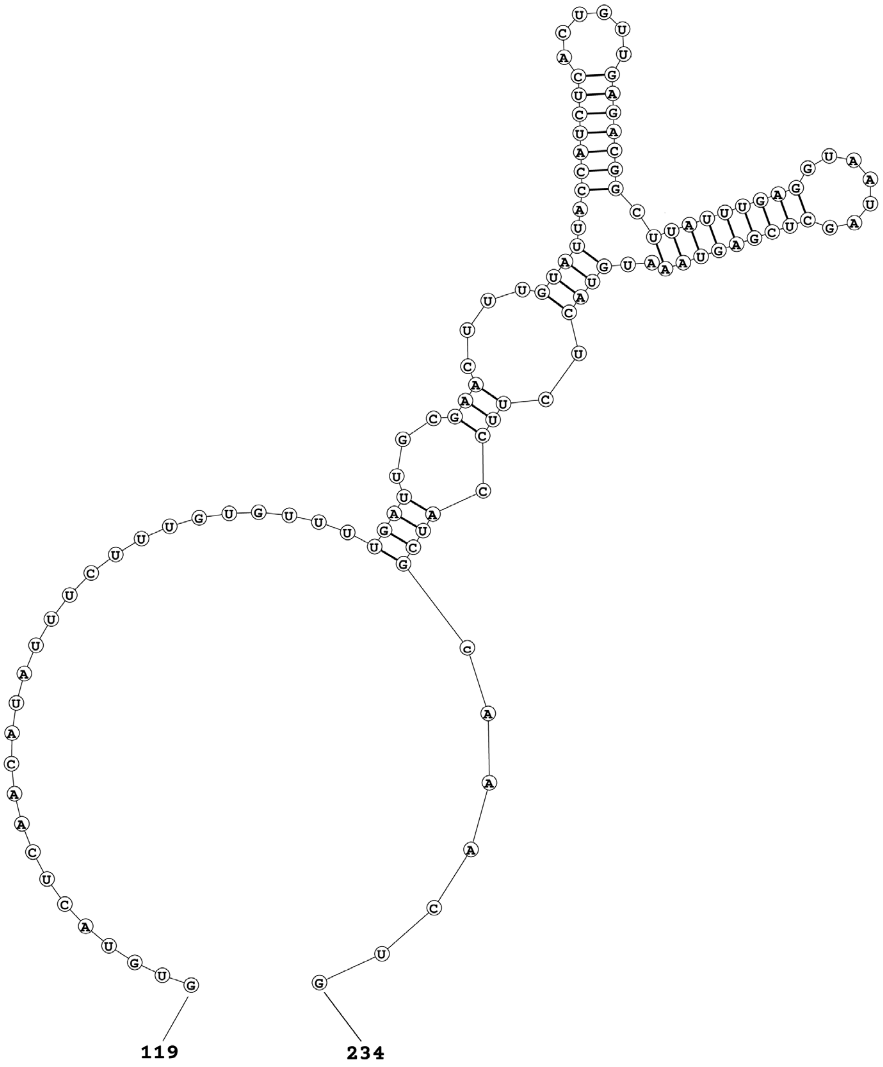

Supplemental Figure S2. Predicted Hooks structure for *RPL28* intron. Numbering is for positions within the 511-base intron.

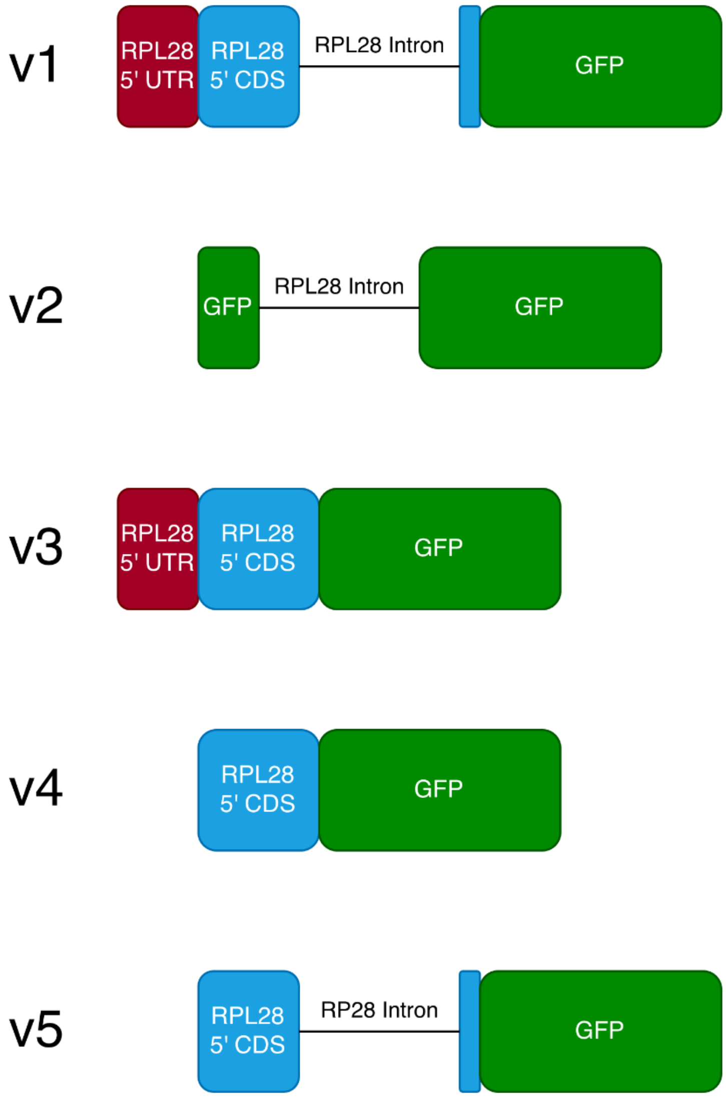

Supplemental Figure S3. Insertions of RPL28 sequences into GFP.

V1: original insert containing 16 bases of the 5' UTR, the first 49 bases of the coding sequence, the intron, and 8 more bases of the coding sequence 5' to GFP.
V2: Inton only inserted into GFP
V3: same as V1 but without the intron
V4: coding region only, no 5' UTR or intron
V5: same as V1 without the 5' UTR

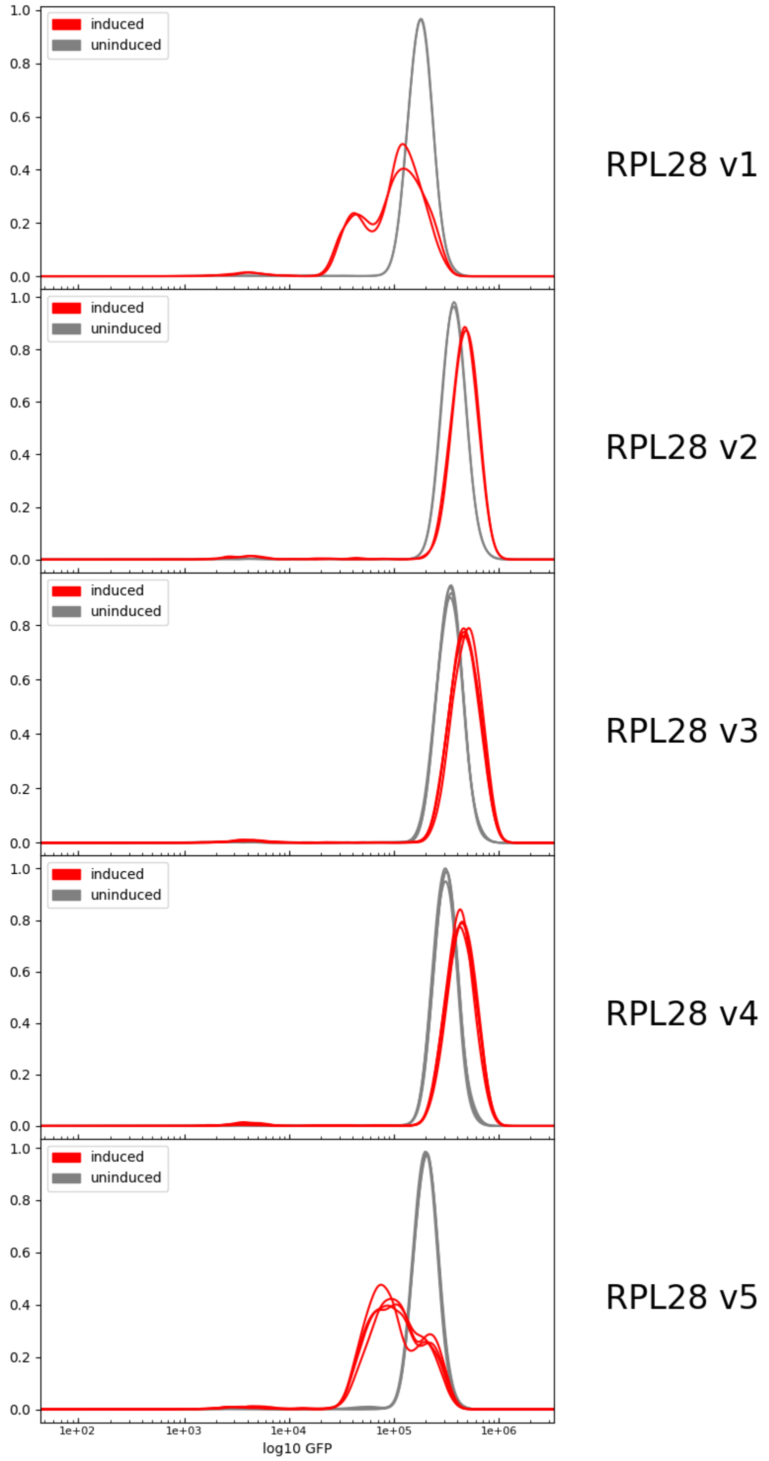

Supplemental Figure S4: GFP expression for insertion variants

Horizontal axis is log of GFP fluorescence, vertical axis is number of cells.

The constructs are labeled as in Figure S1.

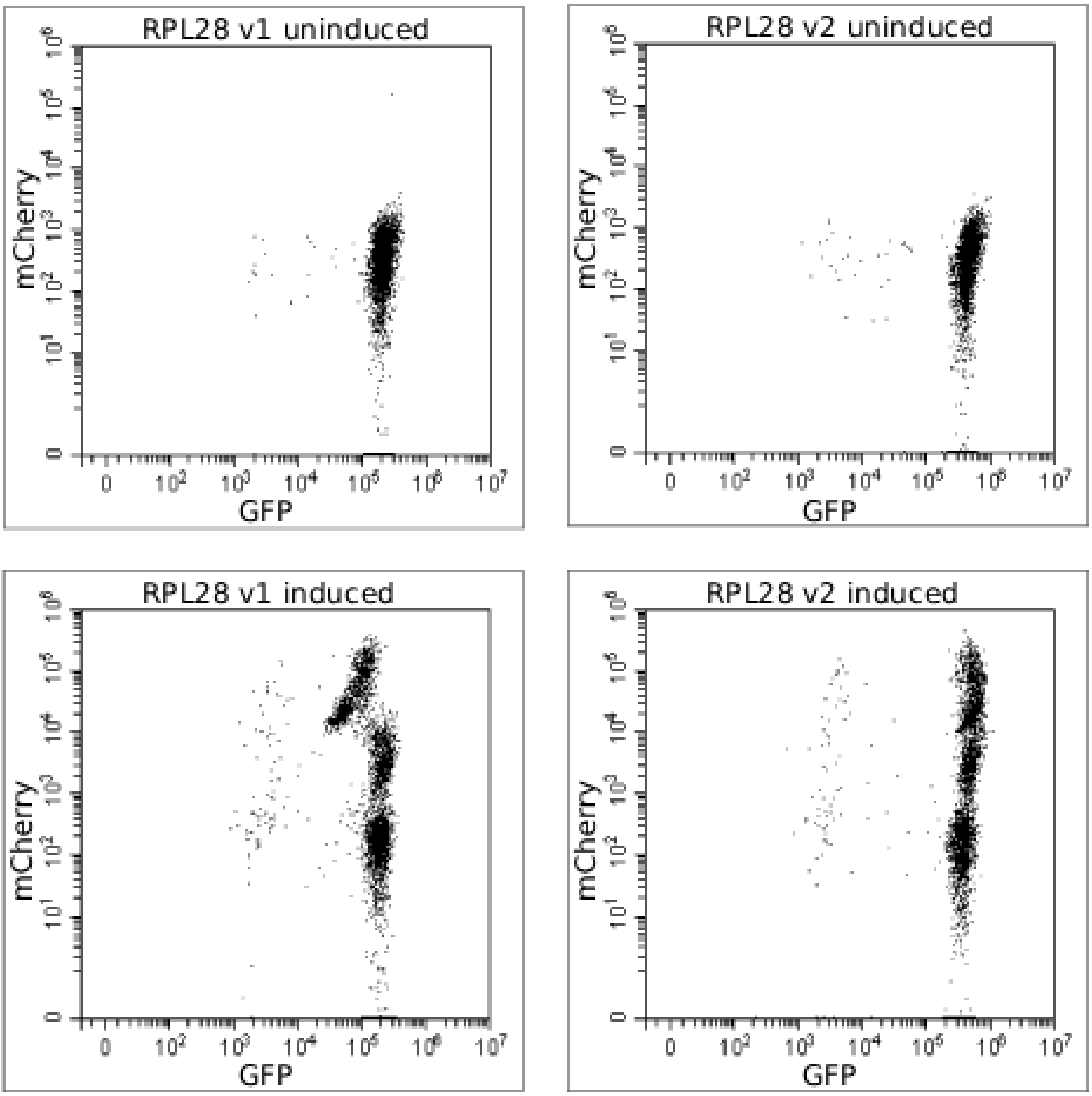

Supplemental Figure S5: 2D plots of GFP and mCherry fluorescence

Each dot is a single cell. Plots show both the GFP (horizontal axis) and mCherry (vertical axis) fluorescence values. Left column plots are for construct V1 and right column plots are for construct V2. Upper row in for uninduced condition and lower row is for induced Rpl28 (tagged with mCherry) condition.
